# Recent Updates Regarding the Antiproliferative Activity of *Galium verum* Extracts on A375 Human Malignant Melanoma Cell Line

**DOI:** 10.3390/life14010112

**Published:** 2024-01-11

**Authors:** Alexandra-Denisa Semenescu, Elena-Alina Moacă, Andrada Iftode, Cristina-Adriana Dehelean, Diana-Simona Tchiakpe-Antal, Laurian Vlase, Slavita Rotunjanu, Delia Muntean, Sorin Dan Chiriac, Raul Chioibaş

**Affiliations:** 1 Department of Toxicology, Drug Industry, Management and Legislation, Faculty of Pharmacy, “Victor Babes” University of Medicine and Pharmacy Timisoara, 2nd Eftimie Murgu Square, 300041 Timisoara, Romania; alexandra.scurtu@umft.ro (A.-D.S.); alina.moaca@umft.ro (E.-A.M.); cadehelean@umft.ro (C.-A.D.); 2 Research Centre for Pharmaco-Toxicological Evaluation, “Victor Babes” University of Medicine and Pharmacy Timisoara, 2nd Eftimie Murgu Square, 300041 Timisoara, Romania; 3 Department of Pharmaceutical Botany, Faculty of Pharmacy, “Victor Babes” University of Medicine and Pharmacy Timisoara, 2nd Eftimie Murgu Square, 300041 Timisoara, Romania; diana.antal@umft.ro; 4 Department of Pharmaceutical Technology and Biopharmaceutics, Faculty of Pharmacy, “Iuliu Hatieganu” University of Medicine and Pharmacy, 8th Victor Babes Street, 400347 Cluj-Napoca, Romania; laurian.vlase@umfcluj.ro; 5 Department of Pharmacology-Pharmacotherapy, Faculty of Pharmacy, “Victor Babes” University of Medicine and Pharmacy Timisoara, 2nd Eftimie Murgu Square, 300041 Timisoara, Romania; slavita.rotunjanu@umft.ro; 6 Department of Microbiology, Faculty of Medicine, “Victor Babes” University of Medicine and Pharmacy Timisoara, 2nd Eftimie Murgu Square, 300041 Timisoara, Romania; muntean.delia@umft.ro; 7 Multidisciplinary Research Center on Antimicrobial Resistance, “Victor Babes” University of Medicine and Pharmacy Timisoara, 2nd Eftimie Murgu Square, 300041 Timisoara, Romania; 8 Department X—Surgery II, Faculty of Medicine, “Victor Babes” University of Medicine and Pharmacy Timisoara, 2nd Eftimie Murgu Square, 300041 Timisoara, Romania; chiriac.sorin@umft.ro; 9 Department IX—Surgery I, Faculty of Medicine, “Victor Babes” University of Medicine and Pharmacy Timisoara, 2nd Eftimie Murgu Square, 300041 Timișoara, Romania; office@medcom.ro; 10 CBS Medcom Hospital, 12th Popa Sapca Street, 300047 Timisoara, Romania

**Keywords:** skin cancer, quality of life, antioxidants, DPPH test, LC-MS, cytotoxicity, phytochemical screening

## Abstract

The biological activity of *Galium verum* herba was exerted on various tumor cell lines with incredible results, but their potential effect on malignant melanoma has not been established yet. Therefore, the current study was structured in two directions: (i) the investigation of the phytochemical profile of diethyl ether (GvDEE) and butanol (GvBuOH) extracts of *G. verum* L. and (ii) the evaluation of their biological profile on A375 human malignant melanoma cell line. The GvDEE extract showed an FT-IR profile different from the butanol one, with high antioxidant capacity (EC_50_ of GvDEE = 0.12 ± 0.03 mg/mL > EC_50_ of GvBuOH = 0.18 ± 0.05 mg/mL). The GvDEE extract also showed antimicrobial potential, especially against Gram-positive bacteria strains, compared to the butanol extract, which has no antimicrobial activity against any bacterial strain tested. The results regarding the antitumor potential showed that both extracts decreased A375 cell viability largely (69% at a dose of 55 µg/mL of the GvDEE extract). Moreover, both extracts induce nuclear fragmentation by forming apoptotic bodies and slight chromatin condensation, which is more intense for GvDEE. Considering the results, one can state that the *Galium verum* herba possesses antitumor effects on the A375 human malignant melanoma cell line, a promising phytocompound for the antitumor approach to skin cancer.

## 1. Introduction

The International Agency for Research on Cancer (IARC) published the GLOBOCAN 2023 database (https://gco.iarc.fr/tomorrow/home, accessed on 5 November 2023) regarding melanoma, which is considered one of the most common skin cancers with an estimated incidence in Europe (150 vs. 172 thousand new cases from 2020 to 2040) and mortality (26.3 vs. 33.4 thousand deaths from 2020 to 2040). In Romania, the incidence of melanoma was 1.55 thousand cases in 2020 for both sexes aged 0–85+ and is estimated to increase until 2040 to 1.56 thousand cases, as well as the mortality, which is assumed to increase from 502 deaths in 2020 to 551 deaths in 2040 [1]. Melanoma is the most aggressive form of skin cancer deriving from melanocytes involved in melanin cell production [2]. Generally, melanoma is developed at the skin level but rarely can occur on mucous membranes (rectum, vagina, and vulva) as well as at the ocular level, comprising the iris, choroid, and ciliary body [3]. Taking into account the tissue origin where melanoma occurs, the tumor form, infiltration, and spreading rate, as well as the metastatic behavior, this specific type of cancer could be classified into the following subtypes: (1) amelanotic melanoma; (2) superficial melanoma; (3) acral lentiginous melanoma; (4) lentigo malignant melanoma; and (5) nodular melanoma, which appear deeper into the skin [4]. The causes of melanoma development are multifactorial; among the most well-known are genetic predisposition (incorrect DNA repair), solar radiation (mainly UVB rays), and existing injuries, which can influence melanoma initiation and promotion [3,5,6,7]. The dominant risk factors reported to be involved in the dramatic increase in skin cancer incidence are ultraviolet radiation overexposure (UV rays) and skin type. In addition to these dominant factors, other factors such as age, gender, geographic area, common or atypical nevi, and genetic inheritance [8,9] contribute to malignant melanoma susceptibility, some of them (personal factors) being attributed to the melanin content in skin layers [10,11,12].

Melanoma can be easily treated when diagnosed in its early stages through surgery [13], but at advanced stages, the treatment becomes very difficult due to the metastases, including brain metastases; thus, in this stage, melanoma becomes resistant to conventional therapy based on anticancer drugs, even radiotherapy [14,15,16]. Therefore, the development of novel therapeutic alternatives that will be effective, safe, and improve clinical outcomes for melanoma, which do not present low response rates [17] or significant toxicity [18,19], is urgently needed. Over the years, several research groups were concerned with determining the antitumor potential of natural product molecules suitable for melanoma treatment, contributing to developing a new anticancer drug. Therefore, the molecules derived from plants, microorganisms, or animals remain an important source for medical formulations with anticancer effects [20,21]. At the moment, the Food and Drug Administration (FDA) approved for clinical purposes several anticancer agents that are directly or indirectly derived from natural sources (vinblastine, vincristine, vinorelbine, paclitaxel, docetaxel, topotecan, irinotecan, etc.) [22,23,24]. However, the demand for the development of new/natural therapeutic anticancer agents that are more effective and have less toxic effects, which can be used individually or in combination with conventional anticancer drugs to produce a synergistic effect in the treatment of melanoma, is continuously increasing [25,26,27]. Because melanoma shows a high level of reactive oxygen species (ROS), therefore, the antioxidant compounds can be considered suitable candidates for the development of alternative therapeutic options. Among antioxidants, polyphenolic compounds, especially flavonoids, are widespread in medicinal plants, possessing a large spectrum of pharmacological properties, including anticancer [28], being characterized by their pregnant antioxidant properties [29]. Flavonoids are bioactive compounds, secondary metabolites of plants that possess anticancer effects, with particular therapeutic interest in the context of skin cancer [24,30]. Thus, medicinal plants and their bioactive compounds have been and continue to be used as complementary alternative therapies.

The genus *Galium* consists of over 670 herbaceous plant species all over the world, most of them in Europe, North Africa, and Asia, as well as the northern half of the United States [31]. It was reported that *Galium* species have been used to reduce infection and inflammation in living organisms and to treat wounds, burns, and skin diseases [31,32]. In the Romanian flora, there are approximately 38 species of *Galium*, most with white flowers. Still, the most known is *Galium verum* L. (a species with yellow flowers), known also as lady’s bedstraw or yellow bedstraw and called sânziana in Romanian folklore [33]. This species has been used for many years in food manufacturing and folk medicine, being a rich source of bioactive phytocompounds such as iridoid glycosides, terpenes, monoterpene glycosides, phenolic acids, flavonoids, anthraquinones, essential oils, and vitamin C [34,35,36,37,38,39]. With regard to food manufacturing, the *Galium verum* L. flowers were used for cheese production, as they cause milk coagulation [40]. *Galium verum* L.—a healing plant belonging to the *Rubiaceae* family—has been used in traditional medicine for many years due to its depurative, diuretic, laxative, antirheumatic, wound healing, oral anti-inflammation, and sedative effects [34,41,42]. In appearance, *Galium verum* L. is a short plant whose stem does not grow more than 100–120 cm long. It has long (1–3 cm) and wide (2 mm) leaves, dark green in color, with hairs on the lower side arranged in spirals. The flowers are yellow with a diameter of 2–3 mm, and they are distributed densely in the form of clusters. In traditional medicine, *Galium verum* L. is widely used for homeopathic purposes (dried aerial parts) and as an exogenous treatment for psoriasis or other skin disorders, including in wound healing. The tea made from this plant was used as a diuretic for the bladder and irritation of the kidney, as well as for the cure of cystitis [43]. The biological and pharmacological studies suggest that *Galium* sp. exhibits antioxidant, anti-inflammatory, antibacterial, antihemolytic, cardio-/hepatoprotector, immunomodulatory, and antiproliferative effects [32,43,44,45,46,47,48,49,50,51]. In cancer therapy, it has been reported that *Galium verum* L. was used as a treatment for tongue, head, and neck cancers, as well as breast and ulcer cancers [43,45]. Related to the aforementioned information, and after thorough bibliographic research in the specialized literature, there are no reports about the in vitro antitumor potential of *Galium verum* L. herba on malignant melanoma. In addition, we believe that the native species of *Galium* from western Romania have not yet been thoroughly characterized and biologically investigated.

Therefore, the purpose of this study was to emphasize the phytochemical profile of the diethyl ether and butanol extracts of the native Romanian *Galium verum* L. species through the determination of their polyphenolic compounds as well as antioxidant capacity. Moreover, to establish their anticancer potential on cutaneous malignant melanoma, an in vitro preliminary biological evaluation was performed, consisting of the determination of the antiproliferative activity of the extracts on human malignant melanoma cells (A375) and human keratinocyte cell lines (HaCaT). In addition, their antimicrobial potential was also determined using both Gram-positive and Gram-negative bacilli strains.

To ensure a high quality of both extracts and to extract polyphenolic compounds with high purity, the *Galium verum* L. herba was purchased from a store with bio-products. We believe that the results obtained in the present study will complete the lack of information in the literature regarding the antitumor potential of *Galium* species on malignant melanoma, considering that the effectiveness of this species on this specific type of cancer has not yet been demonstrated; this also represents the novelty of the current study.

## 2. Materials and Methods

### 2.1. Reagents and Bacterial Strains

The plant material (*Galium verum* herba) was acquired from AdNatura Company (S.C. ADSERV S.R.L, Brasov, Romania, batch no. 11/2022) and maintained in appropriate conditions at a temperature of 22 ± 2 °C until processing.

To obtain the *Galium verum* extracts, the diethyl ether (≥99.0%) and butanol (≥99.5%) were acquired from Merck KGaA (Darmstadt, Germany). The 2,2-diphenyl-1-picrylhydrazyl (DPPH) was acquired from Sigma-Aldrich, Steinheim, Germany, for the antioxidant capacity evaluation. The ascorbic acid (vitamin C), purchased from Lach-Ner (Prague, Czech Republic), was used as a control to compare the results of both extracts. All chemicals used were of high analytical grade purity.

For LC-MS analysis, the standards acquired from Sigma-Aldrich (St. Louis, MO, USA), namely phenolic acids (chlorogenic and 4-*O*-caffeoylquinic acid), as well as flavonoids (rutin, quercetin, quercetol, quercitrin, and isoquercitrin), were used. In addition, the rest of the standards were procured from Roth and Alfa-Aesar (Karlsruhe, Germany), as well as from Merck KGaA (Darmstadt, Germany), namely luteolin, (+)-catechin, and (−)-epicatechin, as well as phenolic acids (gallic, vanillic, syringic, and protocatechuic acids). Methanol and acetic acid were purchased from Merck KGaA (Darmstadt, Germany), and ultrapure deionized water was provided by a MiliQ System from Merck Millipore (Darmstadt, Germany).

The American Type Culture Collection (ATCC) (Manassas, VA, USA) provided the microorganism strains for the antibacterial potential assay. The most representative Gram-positive—*Staphylococcus aureus* (25923™) and *Streptococcus pyogenes* (19615™)—and Gram-negative—*Escherichia coli* (25922™) and *Pseudomonas aeruginosa* (27853™)—bacterial strains were used. The bacilli strains were initially isolated on Columbia agar supplemented with 5% sheep blood acquired from ThermoScientific Company (Waltham, MA, USA). The NaCl solution used for the dilution of the standardized bacterial inoculum was acquired from bioMérieux, Marcy-l’Étoile, France. The rest of the reagents used for the antimicrobial assessment were purchased from ThermoScientific, Waltham, MA, USA.

For the in vitro experiments, Dulbecco’s Modified Eagle’s medium (DMEM) supplemented with glucose (4.5 g/L) and fetal bovine serum (FBS), procured from PAN-Biotech GmbH (Aidenbach, Germany), was used. In addition, a mixture of penicillin/streptomycin (P/S—10,000 IU/mL), trypsin-EDTA solution, phosphate-buffered saline (PBS), and dimethyl sulfoxide (DMSO—solvent) were utilized for the experiments, purchased from Sigma-Aldrich, Merck KGaA (Darmstadt, Germany). The MTT (3-(4,5-dimethylthiazol2-yl)-2,5-diphenyltetrazolium bromide) viability kit was acquired from Roche Holding AG (Basel, Switzerland), and the lactate dehydrogenase (LDH) cytotoxicity kit was procured from ThermoFisher Scientific (Waltham, MA, USA). 

### 2.2. Methods

#### 2.2.1. Extraction Procedure and Extraction Yield Assessment

**Figure 1:**
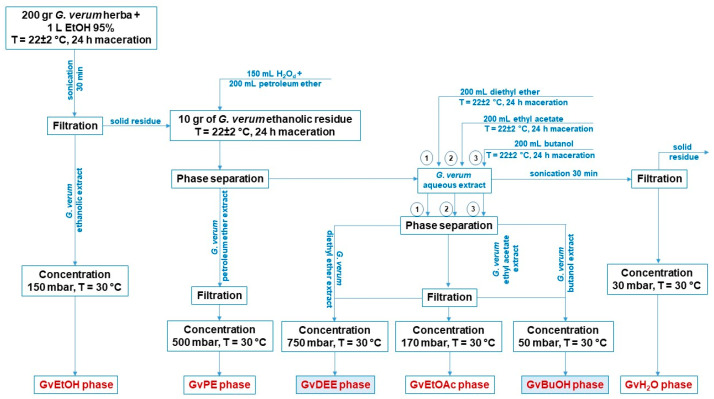
Diagrammatic representation regarding the preparation of *G. verum* L. extracts.

The dried and crushed *Galium verum* herba were subjected to the extraction procedure according to the method of Antoniak and co-workers [52], slightly modified. The extraction procedure consists of obtaining several phases using various solvents, starting with ethanol 95%. Briefly, 200 g of *G. verum* herba was firstly mixed with 1000 mL of ethanol 95% (EtOH 95%), and the mixture was left to macerate for 24 h at 22 ± 2 °C. After maceration, the mixture was sonicated for 30 min using an ultrasonic water bath (ELMA S120 Elmasonic from Elma Schmidbauer GmbH, Singen, Germany), followed by a filtration procedure using Whatman grade 4 filter paper; then, a nylon membrane filter with a 0.45 μm pore size provided by Agilent Technologies (Santa Clara, CA, USA) was used to assure the extract sterilization. Further, the ethanolic phase (GvEtOH) was obtained by concentrating the ethanolic extract using a rotary evaporator—Laborata 4000eco from Heidolph Instruments, GmbH & Co. KG (Schwabach, Germany)—at 150 mbar and 30 °C. After the filtration procedure, from the total solid residue, 10 g were weighed, over which a mixture of 150 mL of distilled water and 200 mL of petroleum ether was added and left to soak for 24 h at 22 ± 2 °C. The next day, phase separation occurred (aqueous phase and petroleum ether phase). The petroleum ether extract was filtered and concentrated at 500 mbar and 30 °C until the GvPE phase was obtained. Sequentially, 200 mL of each solvent, diethyl ether, ethyl acetate, and butanol, was added over the *G. verum* L. aqueous extract. After maceration for 24 h at room temperature, phase separations (aqueous phase and organic phase), filtration, and extract concentration (the parameters are detailed in Figure 1), the GvDEE, GvEtOAc, and GvBuOH phases were obtained. During the maceration process at room temperature (22 ± 2 °C), all the Erlenmeyer recipients with solvent extracts were covered with a parafilm film.

The aqueous phase was further filtered and then concentrated at 30 mbar and 30 °C; thus, the GvH_2_O phase was obtained. All the phases obtained were kept in a refrigerator at 4 °C until further use (characterization and biological assessment). The schematic protocol of the phases obtained is outlined in Figure 1.

In the present study, only the GvDEE (diethyl ether) and GvBuOH (butanol) extracts were of interest. The final volume obtained for each extract was 167 mL for GvDEE and 189 mL for GvBuOH, but the extraction efficiency was calculated only for 50 mL of each extract using Equation (1). The 50 mL of each extract was initially subjected to solvent evaporation at a constant temperature (25 °C) to prevent the degradation of the natural compounds extracted.

(1) $$\eta \left[\%\right]=\frac{m_{concentrate}\times V_{extract}}{V_{total\;extract}\times m_{plant\;material}}\times 100,$$

where η is the extraction yield [%]; $m_{concentrate}$ is the quantity of the concentrate obtained after the solvent evaporation [g]; $V_{extract}$ is the extract volume subjected to concentration step (50 mL) [mL]; $V_{total\;extract}$ is the extract final volume obtained in the extraction procedure [mL]; and $m_{plant\;material}$ is the quantity of the *Galium verum* herba subjected to the extraction process [g].

#### 2.2.2. Polyphenol Screening by FT-IR and HPLC

The Fourier transform–infrared spectroscopy (FT-IR) was employed for the organic functional group identifications present in both *Galium verum* L. extracts. The instrument used was a Prestige-21 spectrometer from Shimadzu Corporation (Duisburg, Germany) based on KBr pellets ranging from 4000 to 400 cm^−1^ with a resolution of 4 cm^−1^. All the measurements were made at room temperature (22 ± 2 °C).

Liquid chromatography coupled with mass spectrometry (LC/MS) was used to identify and quantify the polyphenols present in both extracts. The previously validated described method [53,54,55] was applied using the HPLC Series System coupled with a mass spectrometer (LC/MSD Ion Trap SL from Agilent1100) from Agilent Technologies (Santa Clara, CA, USA). For the phase separation, a reverse-phase analytical column (Zorbax SB-C18) at a working temperature of 48 °C was used. Using the UV and MS modes, the detection of the compounds present in both extracts was performed by setting the parameters described in a previous study [56]. The methanol and acetic acid 0.1% form the binary gradient used as the mobile phase. The first elution (5 μL injection volume of 5% methanol for 35 min) ran at a flow rate of 1 mL·min^−1^, starting with a binary linear gradient and ending at 42% methanol. The isocratic elution lasted 3 min and started with 42% methanol, followed by the column rebalancing for 7 min with 5% methanol [57]. The concentration of the polyphenols was determined according to a calibration curve of the standards, ranging from 0.1 to 50 μg/mL, with good linearity (R^2^ = 0.999), and the results were represented as the μg of polyphenols/mL of *G. verum* extract. In addition, the catechins, epicatechins, and other phenolic acids (gallic, syringic, vanillic, and protocatechuic acids) were investigated.

#### 2.2.3. Antioxidant Capacity

The antioxidant capacity (AC) of *G. verum* diethyl ether and butanol extracts was determined using the DPPH free radical capturing test, according to the previously reported protocol [58]. Six different concentrations were prepared for each extract to evaluate the EC_50_ parameter. The EC_50_ represents the half maximal inhibitory concentration of the antioxidant compounds present in both *G. verum* extracts needed to capture 50% of DPPH free radicals from the test solution. Briefly, the method consists of the preparation of a 0.1 mM DPPH solution in ethanol, which was kept in the refrigerator until further use. Then, 300 μL of each test sample was mixed with 2.7 mL of DPPH 0.1 mM ethanol solution, and the absorbances were read in a continuous mode for 20 min at 517 nm wavelength using the UviLine 9400 spectrophotometer from SI Analytics (Mainz, Germany). For comparison, a control was used based on an ascorbic acid (vitamin C) solution of 0.4 mg/mL in 95% ethanol. For the quantification of the antioxidant capacity, Equation (2) was used:

(2) $$AC\;\left[\%\right]=\frac{{Abs}_{\;free\;radical}-{Abs\;}_{sample}}{{Abs\;}_{free\;radical}}\times 100,$$

where ${Abs}_{sample}$ is the absorbance of the test samples with the DPPH free radical solution and ${Abs}_{free\;radical}$ is the absorbance of the DPPH free radical without the test sample.

The half maximal inhibitory concentration (EC_50_) was determined through linear regression analysis by plotting a curve between the inhibition percentages of the antioxidant capacity (AC%) obtained and the concentrations of each test sample using OriginLab 2020b software.

#### 2.2.4. Biologic Activity (Antimicrobial and Antitumor Effects)

##### Antimicrobial Activity

By determining the minimum inhibitory concentration (MIC) and minimum bactericidal concentration (MBC), the antimicrobial activity of the *Galium verum* extracts (diethyl ether and butanol) was assessed. In agreement with the European Committee on Antimicrobial Susceptibility Testing (EUCAST) and Clinical Laboratory and Standard Institute (CLSI), the assay was performed [59,60,61,62,63]. To obtain approximately 5 × 10^5^ colony-forming units/mL (CFU), the standardized bacterial inoculum was diluted in 0.85% NaCl solution, and then the bacterial mixture (bacterial suspension + test compounds) was added to Mueller Hinton broth. The Mueller Hinton broth was supplemented with blood and β-nicotinamide adenine dinucleotide (β-NAD) for *S. pyogenes*, obtaining various dilutions (30, 15, 7.5, and 3.75 mg/mL). After 24 h of incubation at 35 °C, the MIC value was determined—the lowest concentration without visible growth. After that, 1 µL of suspension from the test tube without visible growth was subcultivated on Columbia agar supplemented with 5% sheep blood, thus determining the MBC value. The MBC value was considered the lowest concentration tested, which killed 99.9% of the bacteria. The experiments were performed in triplicate.

##### Cell Viability Assessment

HaCaT—immortalized human keratinocytes (CVCL_0038, CLS, Eppelheim, Germany)—and A375—human malignant melanoma (CRL-1619™, ATCC, Manassas, VA, USA)—were the two cell lines used in the current study. HaCaT and A375 were grown in Dulbecco’s Modified Eagle’s medium to which fetal bovine serum at a concentration of 10% and a mixture of 1% penicillin/streptomycin antibiotics were added as supplements. The experiments were realized in a humidified atmosphere with 5% CO_2_ and a temperature of 37 °C.

The cell viability was assessed using the MTT method. HaCaT and A375 cells were seeded in 96-well culture plates (1 × 10^4^ cells/well). After cell adherence and adequate confluence, the cells were tested with two extracts of *G. verum* L. (GvDEE and GvBuOH) at concentrations of 15, 25, 35, 45, and 55 μg/mL for 24 h. For in vitro analyses, the extracts were diluted with 0.5% DMSO to a stock solution of 1 mg/mL. After the 24 h stimulation period, a volume of tetrazolium salt solution (10 μL) was added to each well, and then the culture plates were incubated for 3 h. The insoluble crystals formed were solubilized with a buffer for 30 min (100 µL for each well). Control cells were treated with specific growth media. The absorbance was read at 570 nm wavelength using a Cytation 5 device from BioTek Instruments Inc. (Winooski, VT, USA).

##### Cytotoxicity Assay

The cytotoxic action of *Galium verum* L. extracts on A375 tumor cells was investigated using the lactate dehydrogenase test. LDH is a cytosolic enzyme, which is released into the culture medium when the plasma membrane is damaged. Therefore, 10^4^ cells/well were cultured in a 96-well plate and allowed to adhere to the plate overnight. Then, the cultured cells were stimulated with five concentrations (15, 25, 35, 45, and 55 μg/mL) of the two extracts and incubated for 24 h. After 24 h of treatment, 50 µL of each well was redistributed to a new 96-well plate, over which 50 µL/well of the reaction mixture was added and then incubated at room temperature for 30 minutes. Finally, 50 μL of stop solution was added to each well. The absorbances were measured at two wavelengths, 490 and 680 nm, respectively, using a Cytation 5 device from BioTek Instruments Inc. (Winooski, VT, USA).

##### Cell Morphology and Confluence Evaluation

Cell morphology and confluence were analyzed to outline the effect of GvDEE and GvBuOH on HaCaT and A375 cell lines. After 24 h of stimulation with the two extracts, a microscope evaluation of their impact on cells was performed by photographing them under bright field illumination. The pictures were obtained using Cytation 1 from BioTek Instruments Inc. (Winooski, VT, USA). The cell confluence (%) was represented after 24 h of treatment with GvDEE and GvBuOH extracts with the help of the Image Analysis tool from Gen5TM Microplate Data Collection and Analysis Software (version 3.14 for Windows).

##### Nuclear Staining Evaluation

The Hoechst 33342 staining method was applied to highlight the cytotoxic effects of the extracts on the nuclei of human melanoma cells. Cells (1 × 10^5^ cells/well) were grown in 12-well plates and stimulated with each extract using only two concentrations—15 and 55 μg/mL. After 24 h, the cell medium was eliminated and 500 μL of Hoechst solution diluted in PBS (1:2000) was added to each well. After 5–10 min in the dark at room temperature, the staining solution was removed and the cells were washed three times with PBS. Apoptosis was quantified by calculating the apoptotic index, applying the formula described in the study reported by Gag and co-workers [64]. To take fluorescent images at a magnification of 10×, the fluorescence inverted microscope Olympus IX73 (Olympus, Tokyo, Japan) was used.

#### 2.2.5. Statistical Analysis

All the results obtained are expressed as the mean values ± standard deviation (SD) of three independent experiments. GraphPad Prism (GraphPad Software, software version 9.4.0 for Windows, San Diego, CA, USA, www.graphpad.com, accessed on 30 October 2023) was the statistical program utilized in the current study. One-way ANOVA and Dunnett’s multiple comparison post-test, where * *p* < 0.05, ** *p* < 0.01, *** *p* < 0.001, and **** *p* < 0.0001, were carried out to compare the groups. The statistical data obtained regarding the antioxidant capacity and FT-IR investigations were processed with the OriginLab 2021b software (Origin Lab—Data Analysis and Graphing Software, version 9.8.5.212, Szeged, Hungary). To identify and quantify the polyphenols by HPLC analysis, all the chromatographic data were processed using the ChemStation (vA09.03) and Data Analysis (v5.3) software from Agilent (Santa Clara, CA, USA).

## 3. Results

The GvDEE and GvBuOH extracts were subjected to spectroscopic characterization (Fourier transform infrared spectroscopy (FT-IR)) to identify the functional groups from each extract. In addition, the phenolic composition was determined through the LC-MS technique, as well as the antioxidant capacity of the obtained extracts. After that, the biological activity of both extracts on human healthy keratinocyte and malignant melanoma cells was assessed to establish the potential antitumor effect on skin cancer.

### 3.1. Extraction Yield and Polyphenol Screening

The extraction yield was calculated by applying Equation (1) with 50 mL of each extract. The extraction yield for the GvDEE extract was 2.25% and the GvBuOH extract was 1.77%, respectively. One can observe that regardless of the solvent used in the extraction process (diethyl ether or butanol), the extraction yields obtained are approximately similar. We can affirm that, in the present case, the solvent used does not significantly influence the extraction yield.

#### 3.1.1. Polyphenols by FT-IR

**Figure 2:**
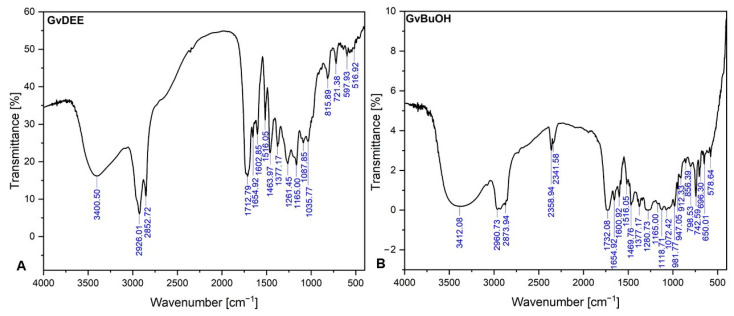
FT-IR spectra of diethyl ether (**A**) and butanol (**B**) *G. verum* extracts.

Considering the match between the absorption bands recorded at a specific wavenumber and, after that, comparing them to the absorption band frequency from the library, we were able to identify the main polyphenols contained in the dried extracts, through the FT-IR qualitative investigation method. The FT-IR spectra of the diethyl ether and butanol extracts of the *G. verum* herba are depicted in Figure 2.

Table 1 reveals the organic functional groups of diethyl ether and butanol extracts as well as the organic bond of each group recorded.

As one can observe from Figure 2 and Table 1, the two extracts (GvDEE and GvBuOH) exhibit absorption bands at similar wavenumbers meaning that specific organic functional groups are present in both extracts, with a few exceptions: the GvBuOH exhibits a doublet-like absorption band at approximately 2300 cm^−1^, which corresponds to the O=C=O stretching functional group from carbon dioxide. Moreover, the same extracts showed at 1118.71 cm^−1^ wavenumber an absorption band which can be attributed to the C–O stretching functional groups from aliphatic ethers or secondary alcohols. Another difference found in the GvBuOH extract was the appearance of multiple bands registered between 912.33 and 981.77 cm^−1^, which can correspond to the =C–H bending functional groups from alkenes or to the C=C bending monosubstituted functional groups from alkanes (the band recorded at 981.77 cm^−1^). The absorption bands recorded at 650.01 cm^−1^ and 696.30 cm^−1^ are present only in the GvBuOH extract and not the GvDEE extract. The first band corresponds to the C–Br stretching functional groups from halo compounds and the second band (696.30 cm^−1^) corresponds to either the =C–H bending functional groups from alkenes or to the C=C bending functional groups, disubstituted from the cis position of the alkenes present in the butanol extract (GvBuOH).

In the case of the GvDEE extract, the exception consists of the appearance of the band registered at 516.92 cm^−1^ corresponding to the C–I stretching functional groups from halo compounds, as well as to the band registered at 1035.77 cm^−1^, which can be attributed to the C–O stretching functional groups from the esters formed after the interaction between an atom of hydrogen (or acidic hydroxyl group) from the solvent with an organyl group from the polyphenols contained in the plant material.

#### 3.1.2. Polyphenols by HPLC

The results regarding the content of polyphenolic compounds of both *G. verum* extracts (diethyl ether and butanol) obtained by LC-MS analysis are presented in Table 2. For each compound, a limit of quantification and detection was imposed (0.1 μg/mL), calculated as the minimal concentration able to produce a reproductive peak with a signal-to-noise ratio > 3.

LC-MS analysis revealed differences between the two extracts, that is, several phenolic compounds were found in *G. verum* diethyl ether extract (eight) and only three phenolic compounds in *G. verum* butanol extract. Chlorogenic acid found in both extracts was detected below the limit of quantification. It can be observed that only two flavonoids were quantified in the GvBuOH extract—isoquercitrin and rutin—while the GvDEE extract contains, in addition to the two flavonoids and quercetol, luteolin and apigenin, as well as phenolic compounds (p-coumaric and ferulic acids). Therefore, the GvDEE extract turned out to be richer in phenolic compounds as well as in flavonoids than the GvBuOH extract. Overall, in both extracts, one can observe a higher amount of flavonoids than phenolic acids. The catechins identified and quantified from both *G. verum* extracts, analyzed by LC-MS, are shown in Table 3.

One can observe that only epicatechin was detected in *G. verum* diethyl ether extract, at a low concentration, and no polyphenols were detected in the GvBuOH extract.

### 3.2. Antioxidant Capacity

**Figure 3:**
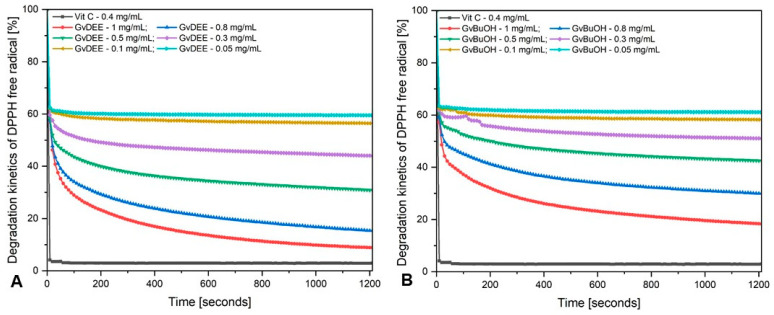
The degradation kinetics of DPPH free radicals are given by the diethyl ether (**A**) and butanol (**B**) *G. verum* L. extracts as well as by the ethanolic solution of control (black line) in a time-dependent manner.

The antioxidant capacity of both extracts is depicted in Table 4, and the degradation kinetics of DPPH free radicals are presented in Figure 3. The antioxidant capacity was evaluated for six different concentrations of each extract to calculate the EC_50_, which represents the concentration of each extract where 50% of its maximal effect is observed, meaning the potency required to obtain a 50% antioxidant effect.

It can be observed that both extracts have shown quite good antioxidant capacity, starting from 38% for the GvBuOH extract and slightly lower in the case of the GvDEE extract, which begins from 40%, at a 0.05 mg/mL concentration. In addition, at 1 mg/mL concentration, the GvDEE extract revealed 91% antioxidant capacity, quite close to the value of the control—Vit C (97%)—at a 0.4 mg/mL concentration.

The degradation kinetics of DPPH free radicals during the period of the evaluation, given by the two extracts are quite different. The GvDEE extract indicates an 8% DPPH free radical degradation at the highest tested concentration (1 mg/mL) as compared with the GvBuOH extract, which indicates approximately 12% degradation, after 20 min of incubation. It is observed that the samples with higher concentrations (1 mg/mL and 0.8 mg/mL) almost quench the DPPH free radicals throughout the evaluation interval, with the reaction reaching equilibrium only after 1000 s. Regarding the samples of 0.5 mg/mL and 0.3 mg/mL concentration, the reaction reaches the equilibrium after 800 s, and the samples with the smallest concentration tested (0.1 mg/mL and 0.05 mg/mL) quench the DPPH free radicals in the first 200 s; subsequently, the reaction reaches equilibrium. In the case of the 0.3 mg/mL concentration of the GvBuOH extract, one can observe some fluctuations in the degradation kinetics of DPPH free radicals, which can be attributed to the small content of polyphenolic compounds present in the butanol extract, as well as the low concentration of the sample tested. Regarding the outcomes obtained, one can affirm that the antioxidant capacity is concentration-dependent for all the samples tested.

### 3.3. Biological Assessment

#### 3.3.1. Antimicrobial Activity

Table 5 presents the results obtained with regard to the antibacterial effect of *G. verum* extracts against Gram-positive and Gram-negative bacilli strains. The antimicrobial potential was investigated by micro-dilution tests evaluating the MIC (mg/mL) and MBC (mg/mL).

The results obtained revealed that only the *G. verum* diethyl ether extract has bacteriostatic and bactericide activities on both Gram+ strains as well as on the *Escherichia coli* strain but does not have bacteriostatic and bactericidal effects on the Gram− *Pseudomonas aeruginosa* strain. Therefore, following the obtained results, we can say that the GvBuOH extract has not exhibited any antimicrobial potential against any of the bacteria strains tested.

#### 3.3.2. Antitumor Activity

##### Viability Assay

The GvDEE and GvBuOH extracts obtained from *G. verum* L. herba were evaluated from an in vitro biological point of view, taking into account one healthy cell line—immortalized human keratinocytes (HaCaT)—and another tumorigenic cell line—primary human skin melanoma A375 cells. Through the MTT colorimetric method, cellular viability was determined. The evaluation was conducted with different extract concentrations, 15, 25, 35, 45, and 55 µg/mL, in the case of both extracts after a stimulation time of 24 h.

**Figure 4:**
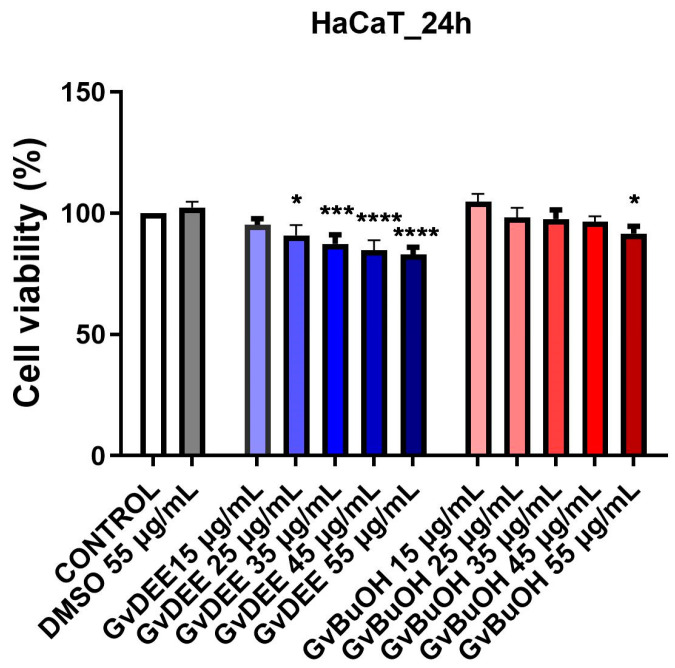
Viability percentage of HaCaT cells after stimulation with GvDEE and GvBuOH extracts at 24 h post-stimulation. Through one-way ANOVA analysis followed by Dunnett’s multiple comparisons post-test, the statistical differences between the control and the treated group (* *p* < 0.05; *** *p* < 0.001; **** *p* < 0.0001) were assessed.

The results obtained regarding the viability of the cells (Figure 4) indicate that both extracts slightly reduced the cell viability of the healthy skin line cells, more significantly in the case of the diethyl ether extract. Therefore, the lowest viability rates were recorded after stimulation of the HaCaT cell line with the diethyl ether extract (GvDEE) at the highest concentration tested (55 µg/mL), with ~83% of viable cells remaining. In the case of the butanol extract of *G. verum* L. (GvBuOH), a slight proliferative effect was observed at the lowest concentration tested (104.7%). In comparison, cell exposure to the highest concentration induces a cell viability percentage of over 90%. Moreover, even the highest concentration of DMSO tested did not have a significant impact on human keratinocytes.

**Figure 5:**
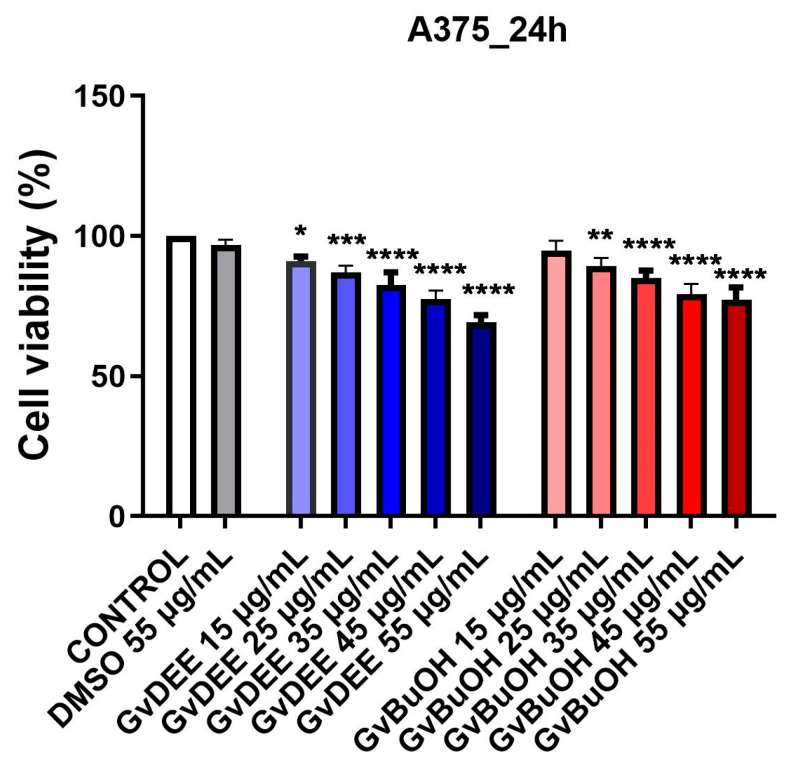
Viability percentage of A375 cells after stimulation with GvDEE and GvBuOH extracts at 24 h post-stimulation. Through one-way ANOVA analysis followed by Dunnett’s multiple comparisons post-test, the statistical differences between the control and the treated group (* *p* < 0.05; ** *p* < 0.01; *** *p* < 0.001; **** *p* < 0.0001) were assessed.

In the case of testing the extracts on the A375 tumor line, the data obtained (Figure 5) showed that both extracts decreased cell viability, with the lowest percentage of viability (69.1%) being observed after exposure to the highest dose of 55 µg/mL of the GvDEE extract for 24 h. In the case of the GvBuOH extract, at the highest concentrations, the cell viability rates were below 80%. Meanwhile, the concentration of 55 µg/mL of DMSO induced an insignificant decrease in the viability of A375 cells. Hence, GvDEE and GvBuOH extracts decreased cell viability in a dose-dependent manner.

Since the MTT method is a colorimetric test for evaluating mitochondrial activity, the release of lactate dehydrogenase was further investigated to ensure that the two extracts have a potential cytotoxic effect on the A375 cell line.

##### *G. verum* L. Extracts’ Cytotoxicity Evaluation

**Figure 6:**
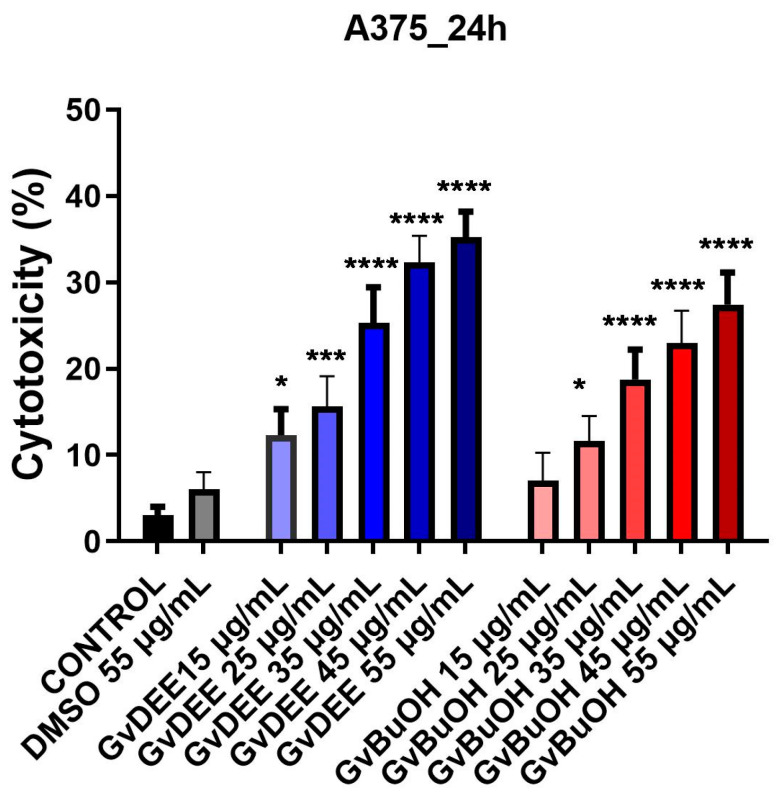
The cytotoxic activity of GvDEE and GvBuOH extracts on the melanoma A375 cell line 24 h post-stimulation. Through one-way ANOVA analysis followed by Dunnett’s multiple comparisons post-test, the statistical differences between the control and the treated group (* *p* < 0.05; *** *p* < 0.001; **** *p* < 0.0001) were evaluated.

After 24 h, it was shown that GvDEE and GvBuOH extracts promoted the release of lactate dehydrogenase in a concentration-dependent manner. The concentration of 55 μg/mL of the diethyl ether extract demonstrated the strongest release of LDH (35.2%) compared to the butanol extract (27.5%). Figure 6 describes the impact of the *Galium verum* extracts on LDH release.

The cytotoxic potential of the two extracts of *G. verum* L. at five different concentrations was determined, and by analyzing the results, an increase in cytotoxic potential with the increasing extract concentrations was observed. Utilizing the LDH assay, it was demonstrated that the GvDEE and GvBuOH extracts have a potential cytotoxic effect on the human melanoma cell line A375.

To outline the anticancer effect of *G. verum* L. extracts, the morphology and confluence of the HaCaT and A375 cell lines were evaluated after exposure for 24 h to several concentrations of the GvDEE and GvBuOH extracts.

##### Cell Morphology and Confluence Evaluation

**Figure 7:**
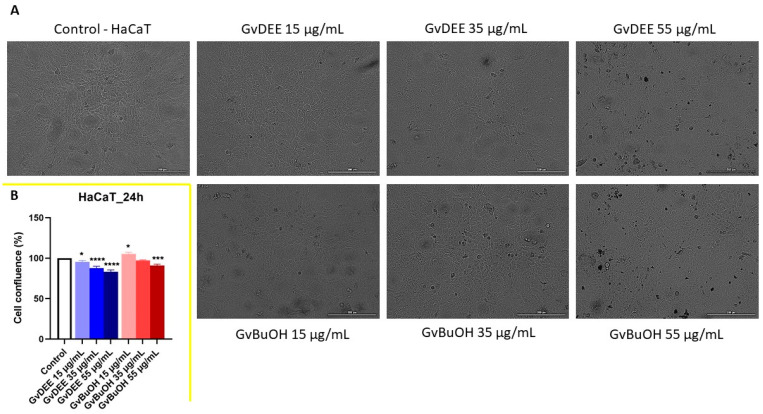
(**A**) The morphological aspect of HaCaT cells after 24 h of treatment with GvDEE and GvBuOH extracts. The scale bars represent 200 µm. (**B**) Cellular confluence (%) of HaCaT cell line after 24 h of treatment with GvDEE and GvBuOH extracts. * *p* < 0.05; *** *p* < 0.001; **** *p* < 0.0001.

Since no significant changes were observed in the decrease in viability at the average doses tested, the confluence and the morphological aspect were further evaluated at the most suggestive concentrations. At the level of human keratinocytes, no morphological changes were evident, only a slight decrease in cell confluence with increasing concentrations, especially for the diethyl ether extract of *G. verum* L. herba (GvDEE extract to ~85%). Still, for the GvBuOH extract, at the lowest concentration, a slight increase in confluence (to ~105%) was observed (Figure 7A,B).

**Figure 8:**
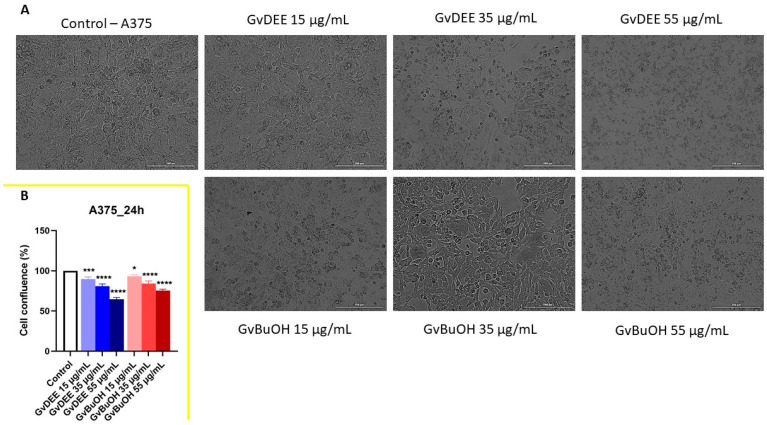
(**A**) The morphological aspect of A375 cells after 24 h of treatment with GvDEE and GvBuOH extracts. The scale bars represent 200 µm. (**B**) Cellular confluence (%) of A375 cell line after 24 h of treatment with GvDEE and GvBuOH extracts. * *p* < 0.05; *** *p* < 0.001; **** *p* < 0.0001.

Regarding human melanoma cells, the GvDEE extract showed the strongest decrease in their confluences, with changes regarding the cell’s shape. The cells were shrunken and rounded after treatment with GvDEE extract, leading to their detachment from the plate, even when low concentrations of the extract were used (35 µg/mL), but especially when the cells were treated with the highest concentration (55 µg/mL, to ~65%). The GvBuOH extract induced the reduction in confluence with doses increasing but in a lower percentage (to ~75%) than the GvDEE extract, noting that the cells are stressed, round, and shrunken, as shown in Figure 8A,B.

##### Nuclear Staining Evaluation

**Figure 9:**
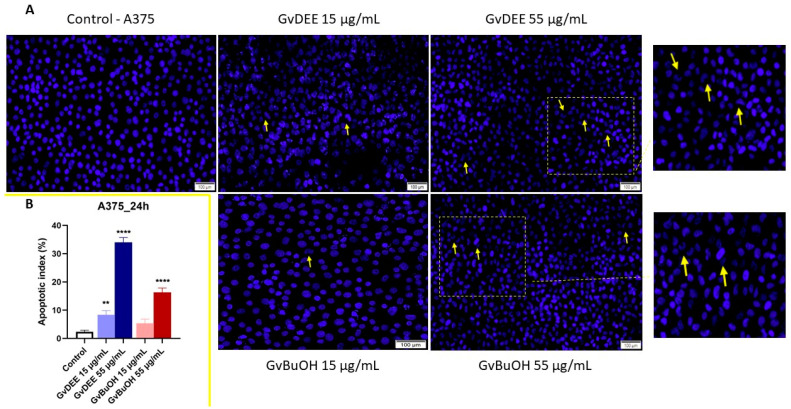
(**A**) The morphological changes at the nuclear level after 24 h of treatment with GvDEE and GvBuOH extracts (15 and 55 µg/mL). The yellow arrows highlighted the apoptosis signs. The scale bars indicate 100 µm. (**B**) Apoptotic index determination in A375 cells marked through Hoechst staining test after 24 h of treatment with GvDEE and GvBuOH extracts. ** *p* < 0.01; **** *p* < 0.0001.

Observing the low viability rates of A375 cells at the highest concentration tested, 55 µg/mL, we have agreed to investigate the potential morphological changes that may occur at the nuclear level after 24 h of treatment with the GvDEE and GvBuOH extracts using the Hoechst 33342 staining test. Moreover, in parallel, the impact of the lowest concentration of the extracts on the nucleus of A375 cells was visualized. Nuclear deformations provide reliable data on the cytotoxic effect of a compound and indicate the pattern of cytotoxicity (apoptosis or necrosis). The nuclei of cells in the control group are round and regular in shape, and no signs of nuclear fragmentation were observed. Compared with the nuclei of untreated cells, typical features of apoptosis-like cell death were captured by fluorescence microscopy in the nuclei of cells stimulated with the highest concentration of extracts and less at the concentration of 15 µg/mL. The two extracts induced nuclear fragmentation and the formation of apoptotic bodies with slight chromatin condensation; these characteristics were more significant in the GvDEE extract (Figure 9A). A significant increase in the apoptotic index (to 34% for the GvDEE extract and 16.3% for the GvBuOH extract at a dose of 55 µg/mL) was observed compared to the control (Figure 9B).

## 4. Discussion

The treatment options for metastatic melanoma have developed quite a lot in recent years, being directly influenced by the disease stage at the diagnosis time and the metastases grade. Currently, the therapy applied is based on chemotherapy, immunotherapy, and surgical intervention [65,66]. Although chemotherapy used immediately after the surgical intervention represents the most applied therapeutic option, especially in the case of those patients who show resistance to immunotherapy and targeted therapy [67], the use of this conventional method implies the treatment’s lack of response and the occurrence of adverse reactions. Therefore, an effective targeted treatment, which includes natural anticancer compounds that will be safe, non-toxic, and biocompatible with the human body and do not produce adverse reactions, is more and more needed. The treatment alternatives, which have involved natural phytocompounds, are rated to be the safer chemotherapeutic agents that exhibit both therapeutic efficacy and selective anticancer activity. Among the natural phytocompounds of a medicinal plant, polyphenolic compounds and some flavonoids have already demonstrated their efficacy in the modulation of signaling pathways in the carcinogenesis process—an important aspect in the context of skin cancer [68,69,70,71,72]. Hence, the present study aims to report an exhaustive overview of the therapeutic effects of natural phytocompounds found in two extracts of *G. verum* herba and the potential effectiveness of this medicinal plant as an alternative treatment against skin cancer—a potential effectiveness that has not yet been proven.

Therefore, the first proposed objective was to obtain extracts based on various solvents from the *Galium verum* L. plant material, which would contain as many bioactive natural compounds as possible. In the extraction process, the type of solvent used is very important because it affects the quantity and quality of the extracted bioactive compounds based on their different properties and interactions with the extracted bioactive compounds, thus influencing the efficiency of extraction. In the present study, the selection of solvents was made based on their purity and availability, as well as safety of use regarding the health and the environment. In addition, several research groups have implemented some guidelines regarding solvent choice in the extraction process with an acceptable ecological level [73,74,75,76]. The solvents commonly used in the extraction process of medicinal plants are either polar solvents (water or/and alcohols), medium polarity solvents (acetone or dichloromethane), and non-polar solvents (chloroform, ethers, hexane). Organic solvents with low polarity are usually chosen as the organic extracting solvent because they have limited solubility in water and high volatility. For this reason, diethyl ether was selected as the extraction solvent, but also for the fact that it is capable of extracting a wide range of organic compounds, is easily distilled or vaporized, and is environmentally friendly due to its low solubility in water; thus, no significant toxicity against aquatic organisms is expected from it [77]. Over time, butanol has been used as an extracting solvent, especially for hydrophilic compounds (e.g., acids), because it obtains high extraction yields and shows good selectivity compared to other solvents [78]. But in the present days, its use is increasingly rare. Compared to other alcohols, butanol has the least water solubility due to its larger surface area of the alkyl chain, which is a hydrophobic component. Thus, its positive rating as a green solvent with low toxicity and safety makes butanol an excellent choice for the extraction of organic polar compounds from plant materials [79,80,81,82]. In addition to the solvent used and the quality of the plant material, the extraction technique employed and the reaction parameters set (duration, temperature) also have an important role in obtaining good extraction yields. In the current study, *Galium verum* L. herba purchased from a natural store was chosen for use instead of collected herba to avoid the plant material having any traces of heavy metals or toxic compounds coming from the growing soil of the plant. Therefore, by applying the classical extraction technique based on maceration overnight, sonication for several minutes, filtration, and concentration, the GvDEE and GvBuOH extracts were obtained with extraction yields of 2.25% for the diethyl ether phase and 1.77% for the alcoholic phase, calculated from 50 mL of extract. It is observed that the solvent used for the extraction of polyphenolic compounds influences the extraction yield. Compared to the studies reported in the specialized literature, it seems that both the extraction technique and the plant/solvent ratio used significantly influence the extraction yield [56,83,84]. Our results are lower than the results of other studies reported, but the differences can be related to the quality of the plant material as well as the solvent used [85,86].

The phytochemical profile of both *Galium verum* L. extracts was investigated in terms of polyphenolic compounds through LC-MS analysis and FT-IR spectroscopy. Through LC-MS analysis, both phenolic acids and flavonoids were identified in both extracts (Table 2). With regard to the GvDEE extract, the outcomes obtained highlighted the identification of eight polyphenolic compounds, from which only seven were quantified; one phenolic acid (chlorogenic acid) was below the detection limit. Regarding the alcoholic extract (GvBuOH), the results obtained highlighted only three polyphenols, from which two were quantified. Likewise, in this case, from a quantitative point of view, chlorogenic acid was below the detection limit. Quercetol and luteolin are the flavonoids found in high quantities in the GvDEE extract, as compared with rutin, which was higher in the GvBuOH extract than the GvDEE extract. Isoquercitrin was found in similar quantities in both extracts (2.970 μg/mL in the GvDEE extract vs. 2.508 μg/mL in the GvBuOH extract). The remaining polyphenolic compounds from the GvDEE extract were found in low quantities, below 1 μg/mL (p-coumaric acid, ferulic acid, rutin, apigenin, and epicatechin). Through the LC-MS phytochemical analysis, one can separate, detect, and quantify the bioactive plant compounds with good, well-known properties such as antioxidant, anti-inflammatory, and antitumor effects. Therefore, our results are consistent with those reported in the literature, of course with the differences regarding the amount of identified phytocompounds but also their identity [33,87,88], differences that are related to the geographical area where the plant was collected, the extraction technique applied, and the solvent used in the extraction process. In addition, the FT-IR spectroscopic analysis was performed to investigate if the *Galium verum* L. extracts have polymers or inorganic, additive, or contaminant substances in their composition through the identification of functional group fingerprints [89]. The FT-IR analysis revealed the chemical structures of the polyphenolic compounds (Table 1) identified also through LC-MS analysis, perhaps because the phenolic acids and flavonoids from *Galium verum* L. extracts are strong antioxidant compounds and their biologic ability depends on their chemical structure more than their weight in the concentrated extract [90]. Therefore, the most important bands recorded in both extracts were approximately at 3400 cm^−1^ and corresponded to the O–H stretching functional groups from alcohols, phenols, and carboxylic acids [91]. These bands are due usually to the presence of flavones (rutin). The bands recorded between 2850 and 2960 cm^−1^ correspond to the C–H stretching functional groups from alkanes (revealing the presence of the aromatic ring as well as the attachment of the alkyl molecules). In addition, these bands also can correspond to the O–H stretching functional groups from phenolic acids (i.e., chlorogenic, p-coumaric, and/or ferulic acids) due to the broadband intensity recorded in the case of the GvBuOH extract. It was reported that the band recorded at 2926.01 cm^−1^ could be attributed to the CH_3_ vibrations of chlorophyll functional groups from the *Galium verum* L. herba [92]. Peaks recorded around 1700 cm^−1^ indicate the presence of C=O stretching functional groups from α,β-unsaturated esters, aliphatic ketones, or carboxylic acids in the case of the GvDEE extract or of functional groups from esters, δ lactones, or aldehydes in the case of the GvBuOH extract. The bands recorded in both extracts approximately at 1600 cm^−1^ correspond to C=C stretching vibration groups, highlighting the presence of cyclic structure from alkenes, or to the C=O stretching functional groups from primary amides or δ lactames. Bands recorded in both extracts at 1516.05 cm^−1^ correspond to the C=C stretching functional groups from aromatic compounds; these bands confirm the presence of aromatic rings in both extracts [93]. The 1377.77 cm^−1^ wavenumber indicates the presence of phenols, alcohols, or carboxylic acids, which are recorded through the O–H bending functional groups. The structure of butanol is characterized by the O–H groups which form cyclic and linear networks in which the molecules interact with each other [94,95,96]. At approximately 1200 cm^−1^, the bands recorded correspond to the C–O stretching groups from aromatic esters, ethers, acids, and/or to the alkyl aryl ether molecules, especially from the GvDEE extract [97]. These bands could highlight the presence in both extracts of flavonoids/flavones due to their ester-like functional groups, i.e., rutin as well as chlorogenic acid, since it is the ester of caffeic acid. Both extracts also have primary, secondary, and tertiary alcohols in their composition (i.e., the GvBuOH extract and the GvDEE extract have only primary and tertiary alcohols), highlighted by the presence of bands recorded between 1000 and 1100 cm^−1^ [97]. The last region of spectra (500–850 cm^−1^) is attributed to the out-of-plane stretching vibration given by the halo compounds (C–Cl, C–Br, and C–I groups), but, at the same time, it can be attributed to the bending vibration of C=C groups contained by the aromatic compounds (e.g., alkanes in the case of the GvDEE extract) or the bending vibration of =C–H functional groups from alkenes (in the case of the GvBuOH extract). These bending vibration functional groups could indicate the presence of aromatic bicyclic monoterpenes [98]. Considering that the *G. verum* L. herba was purchased from a natural store, we believe that the presence of C–Cl, C–Br, and C–I functional groups could come from the growing soil of the plant or the impurities transferred during the technological process of plant processing. However, in the same manner, the inorganic compounds could have been transferred during the extraction process, respectively, during characterization.

To survey the antioxidant capacity of a plant extract, the stable 2,2-diphenyl-1-picrylhydrazyl radical (DPPH) method is the easiest, most rapid, cheapest, and most sensitive method to apply [99]. The DPPH free radical is a stable violet radical that changes its color to yellow following a reduction process (hydrogen or electron donation). The substances capable of performing the reduction reaction are considered antioxidants, meaning radical scavengers [100]. The capacity of both extracts to scavenge DPPH was determined and the outcomes are shown in Table 4. It has been shown that the diethyl ether and butanol extracts obtained from *Galium verum* L. herba reduce the stable free radical DPPH· to a DPPH–H radical, reaching 50% reduction with an EC_50_ of 0.12 ± 0.03 mg/mL for the GvDEE extract and EC_50_ = 0.18 ± 0.05 mg/mL for the GvBuOH extract. Lakić and co-workers [85] found lower values of inhibition concentrations for the methanol extract of *Galium verum* L. material. The authors reported an IC_50_ between 3.10 μg/mL and 8.04 μg/mL for the alcoholic extract depending on the geographical area of the plant. Another study conducted by Friščić et al. [86] reports an IC_50_ of 30.72 μg/mL for 80% *Galium verum* L. methanol extract. Also, other research studies report lower values compared with ours for the antioxidant capacity of *Galium* sp. when applying the DPPH radical method [101,102,103]. The differences observed between our result and those previously reported can be attributed to the concentration of DPPH used, the reaction conditions, the geographical zone, the age of the plant, the date of collection, the type and volume of solvent, the plant material/solvent ratio used, as well as the extraction technique and parameters set. Almost identical results were obtained by Vlase and co-workers [54] who reported a half maximal inhibitory concentration of 105.43 μg/mL for the 70% *Galium verum* L. ethanolic extract. As in our case, the results obtained may be the consequence of quercetol instead of the other polyphenols recorded in low quantities by LC-MS analysis in the case of the GvDEE extract. Our findings are consistent with the statements made by Li et al. [104], which affirmed that quercetin (sophoretin or meletin or xanthaurine or quercetol [105]) showed better activity than isoquercitrin in an H-donating-based DPPH radical scavenging assay. This is a particularly relevant statement considering that quercetin is present mainly in *O*-glycosidic forms including rutin (as quercetin-3-*O*-rutinoside), isoquercitrin (as quercetin-3-*O*-glucoside), and quercetin-3,4′-*O*-diglucoside [106]. Xiao and his work group [107] reported that in a cell-free assay, isoquercetin showed decreased antioxidant capacity as compared to quercetin dissolved in phosphate-buffered saline (PBS) but greater DPPH capacity when quercetin was dissolved in methanol. Park and co-workers also made the same observations [108] when they used the same flavonols. Hence, through a standard comparison (using the ascorbic acid vitamin C), the antioxidant capacity of the GvDEE and GvBuOH extracts was assessed. One may affirm that by correlating our results with the results reported previously, the health benefits of the *Galium verum* L. plant material were completed, but with regard to the promising future properties related to the mechanism of action in curing skin cancer, complex in vitro as well as in vivo studies need to be performed with more specific and accurate investigation assays.

Because both extracts of *Galium verum* L. plant material contain phenolic acids as well as flavonoids, a class of phytocompound with proven potent antimicrobial effects, the antimicrobial activity was determined through a disc-diffusion assay. Two Gram+ bacteria (e.g., *Staphylococcus aureus* and *Streptococcus pyogenes*) and two Gram− bacteria (e.g., *Escherichia coli* and *Pseudomonas aeruginosa*) were included in the panel of microorganisms used because these bacterial strains were involved in skin pathology. Our results showed that the GvDEE extract was effective against Gram-positive bacilli strains, but against Gram-negative bacilli strains, it was effective only against *Escherichia coli*. Against *Pseudomonas aeruginosa* strains, the GvDEE extract has neither bacteriostatic nor bactericidal activity. With regard to the GvBuOH extract, it did not show antimicrobial activity on any of the tested bacterial strains (Table 5). Therefore, one may affirm that considering the results obtained, it seems the only biologically active compounds involved in the antimicrobial activity were p-coumaric and ferulic acids (phenolic acids), as well as quercetol, luteolin, and apigenin, because those were the phytocompounds present in the GvDEE extract when compared with the GvBuOH extract. None of the extracts showed antimicrobial activity on the Gram-negative bacterial strain used (*Pseudomonas aeruginosa*); this result can be explained by the fact that this bacterial strain is more resistant because it has a complex structure of the bacterial wall and is resistant to numerous antibiotics. However, our results contradict the results reported by Shynkovenko et al. [109], which affirmed that the 96% *Galium verum* L. ethanol extract presents an increased antimicrobial effect against the *Pseudomonas aeruginosa* Gram-negative strain. According to Vlase et al. [54], who stated that the type of solvent might influence the antimicrobial activity, the differences may be due to the type of solvent used; in the current study, butanol ≥ 99.5% was used instead of ethanol 96%. In addition, Ilyina and co-workers [110] reported a significant level of antimicrobial activity of *Galium verum* L. chloroform extract, thus completing Vlase’s affirmation. Therefore, it seems that the Gram-positive bacilli strains were more sensitive when in contact with *Galium verum* L. diethyl ether extract, probably because they have cell walls with a single layer. Our observations are in agreement with the literature [111,112].

The excessive formation of reactive oxygen species (ROS) as well as free radicals is involved in the pathogenesis of multiple disorders at the cellular and tissular levels, leading to various diseases such as inflammatory, cardiovascular, respiratory, neurologic, diabetes, and cancers [113]. For the primary prevention of health problems and the diseases listed above, the most important approach consists of the minimization of oxidative stress. Thus, compounds with antioxidant activity can defend the organism from oxidative stress caused by the uncontrolled formation and excessive release of ROS, lipid peroxidation, protein damage, and breaking of the DNA chain [114,115]. Antioxidants are present in high quantities in fruits, vegetables, and plant materials. Plants remain the cheapest source of natural antioxidants used also for medicinal purposes due to the high amount of phytocompounds (phenolic acids, and/or flavonoids) capable of capturing free radicals by oxidative stress inhibition [116,117]. In traditional medicine, plants were used for drug development and, nowadays, are still used in raw material production for various formulations, supplements, or galenic preparations containing phytochemicals from plant extracts [118]. Through the in vitro investigation methods, the biological activity of the phytocompounds from plant material can be performed, alongside their action mechanisms within the human body. Polyphenols have a huge contribution to organism defense against pathogens as well as in skin protection against ultraviolet radiation (UV). After skin exposure to UV radiation, ROS generation is increased, followed by an excessive release of ROS and then cell inflammation, thus leading to skin cancer. It was stated that flavonoids might have a significant role in ameliorating or even preventing ROS-induced skin damage [119]. Therefore, the last objective proposed in the current study was the assessing of the biological activity of the *Galium verum* L. extracts obtained against human malignant melanoma cells and on immortalized human keratinocytes.

The in vitro assays conducted in the current study denoted that only the *G. verum* extract based on diethyl ether (GvDEE) exerted a slightly decreased cellular viability on healthy cells (but only at the highest concentration tested—55 μg/mL), while the butanol extract (GvBuOH) showed a slightly proliferative effect at the low concentrations tested. At the highest concentration tested (55 μg/mL), this extract (with a low amount of polyphenol content) induces a cell viability percentage of over 90%, but even so, this value corresponds to a non-cytotoxic effect given by GvBuOH on HaCaT healthy cells line. On human malignant melanoma cells, the cytotoxicity given by GvDEE extract was induced starting at the lowest concentration tested (15 μg/mL), with the cytotoxic effect increasing progressively with increasing extract concentration. In the case of the GvBuOH extract, the cytotoxic effect on A375 cells was less significant compared to the GvDEE extract, but still occurred in a dose-dependent manner; almost 20% of the total number of melanoma cells was affected by the antitumor activity of the GvBuOH extract. Moreover, the LDH assay provided an assessment of cell injury resulting from the release of the lactate dehydrogenase enzyme after cell membrane rupture that supports the presence of cytotoxic action. Therefore, it was observed that the highest cytotoxic effect was observed at the concentration of 55 μg/mL by the GvDEE extract, which also produced a significant decrease in cell viability on A375 tumor cells. Based on the results obtained, one can state that the active principles of *G. verum* L. extracts exert antitumor activity by reducing the viability of human malignant melanoma cells at concentrations higher than 55 μg/mL as well as the effect of apoptotic-like activity through the formation of apoptotic bodies.

Considering the results obtained and the fact that the GvDEE extract showed cytotoxicity on human malignant melanoma cells at the lowest concentration tested, one may affirm that, probably, the flavonoids found in this composition exert antitumor activity, namely quercetol, luteolin, and apigenin, more than phenolic acids (p-coumaric and ferulic acids). This observation was also made based on the literature reports. For instance, the research group conducted by Piantelli [120], as well as the one led by Loizzo [121], affirmed that treatment with quercetin leads to the cell proliferation inhibition of various tested human melanoma cells (MNT1, M10, M14, C32, and A375 cells). In addition, it was reported that quercetin induces apoptosis in human melanoma Mel-Juso and G361 cells [122]. In the study effectuated by Cao and co-workers [123], the authors investigated the antitumor activity of quercetin on human melanoma cells (A375, and A2058), as well as on murine melanoma cells (B16F10), and showed that quercetin induces apoptosis in melanoma cells and decreases cell proliferation, migration, and invasion through the downregulation of STAT3 signaling and its targeted gene expression. Moreover, the same compound showed in vivo anti-human melanoma activity, exerting inhibitory effects on tumor growth and metastasis. Other research groups [124,125] reported that flavonoids (like quercetin and luteolin) exhibit considerable antiproliferative activity on different human melanoma cells (OCM-1 and SK-MEL-2), reporting an IC_50_ value ranging from 4.7 to 19 μM. The same authors have investigated the effects of different flavonoids on cell proliferation and cell cycle distribution and demonstrated that the presence of quercetin and luteolin in the chemical structure of the O–H group attached at the ring 3′ position led to an improved cytotoxic effect on human melanoma cells.

With regard to luteolin, the majority of the research studies refer to its impact on pigment synthesis, but this aspect is not related to the therapy or prevention of melanoma [27,126], although an IC_50_ of 115 μM was reported on the A375 melanoma cell line when luteolin was used in a preliminary test [27]. On B16F10 melanoma cells, the biological effect of luteolin was only moderately cytotoxic compared with A375 cells [127], although it was reported that luteolin protected H_2_O_2_-treated HMB-2 melanoma cells [128]. Schomberg and his group of researchers [129] reported that the administration of luteolin on human malignant melanoma leads to the inhibition of cell proliferation by creating a global transcriptomic profile for both treated and untreated tumor cell lines via RNA sequencing. The authors stated that it might be possible that luteolin acts on different pathways that are not associated with oxidative stress to inhibit melanoma cell proliferation, meaning that the biological activity of luteolin is not dependent on its redox modulation ability and, specifically, ROS induction [129].

With regard to apigenin, it was demonstrated that only a dose of 50 μM of apigenin inhibits cell growth of A375 and A2058 human melanoma through cell cycle arrest and apoptosis, and after 24 h, the number of human melanoma cells decreases in a dose-dependent manner [130]. Spoerlein and co-workers [131] reported analogous results when evaluating the cytotoxic potential of apigenin on 518A2 human melanoma cells regarding the cell cycle distribution.

Although the biological activity in vitro and in vivo on different tumor cell lines, including melanoma, has already been demonstrated for each polyphenol presented above, future research studies are necessary regarding the biological activity of these compounds from natural sources (such as from herbal extracts). The current study represents an attempt to investigate the antiproliferative and apoptotic potential of diethyl ether and butanol extracts from *Galium verum* L. plant material on A375 cell lines. The results obtained are insufficient to accurately establish the antitumor potential of the *Galium verum* L. extracts on human malignant melanoma cells; therefore, future studies regarding their in vivo mechanism of action are needed. Therefore, as a future direction of research, we propose to optimize the extraction process using solvents with a strong capacity to capture the bioactive agents from the plant extract, respectively, their isolation, identification, and investigation in vitro and in vivo on human malignant melanoma; thus, the quality of life of patients who are affected by this specific disease will improve significantly.

## 5. Conclusions

The current study represents the first attempt regarding the biological evaluation of two extracts (diethyl ether and butanol) obtained from *Galium verum* L. plant material on human malignant melanoma, which is considered a rare pathology and the main cause of death worldwide. For this purpose, the phytochemical screening and biological profile were investigated. Through the LC-MS investigation, the presence of polyphenolic compounds in *G. verum* L. extracts was identified predominantly more qualitatively and quantitatively in the GvDEE extract. Chlorogenic acid, isoquercitrin, and rutin were the common polyphenols found in both extracts, but the GvDEE extract also exhibited the presence of p-coumaric and ferulic acids, as well as quercetol, luteolin, and apigenin. With regard to the antioxidant capacity, it was found that the DPPH free radical scavenging activities of the GvDEE and GvBuOH extracts increased with increasing extract concentration, with the GvDEE extract having an antioxidant capacity more prevalent than the GvBuOH extract and almost comparable with the standard (vitamin C) at the highest tested concentration (1 mg/mL). The diethyl ether extract exhibits high antimicrobial potential against the Gram + bacterial strains used (*Staphylococcus aureus* and *Streptococcus pyogenes*) and moderate antimicrobial activity against the *Escherichia coli* Gram-negative strain compared with the butanol one, which showed neither a bacteriostatic nor bactericidal effect on any of the strains used. In the present study, the cytotoxic effects of *G. verum* L. on human skin cancer cells were proved, even at low tested concentrations (55 μg/mL). The results exhibit a dose-dependent cytotoxic effect against malignant melanoma cell lines, with more intense activity shown by the diethyl ether extract (GvDEE).

Considering the lack of information regarding the anti-melanoma effect of *Galium verum* L. plant material, one can affirm that the results obtained in the current study complete the gap in the specialized literature. Nevertheless, further investigations of individual compounds and their in vitro/in vivo activities are needed to establish which polyphenolic compounds have a greater anti-melanoma effect.

## Figures and Tables

**Table 1:** Peak values and functional groups of *G. verum* extracts recorded.

GvDEE (*G. verum* Diethyl Ether Extract)		GvBuOH (*G. verum* Butanol Extract)	
Wavenumber [cm^−1^]	Bond Founded in GvDEE Extract/ Functional Groups	Bond Founded in GvBuOH Extract/ Functional Groups	Wavenumber [cm^−1^]
3400.50	O–H stretching (intermolecular bonded) from alcohols	O–H stretching (intermolecular bonded) from alcohols	3412.08
2926.01	C–H stretching from alkanes	C–H stretching from alkane or O–H stretch from alcohols (acid) due to the band intensity (broadband)	2960.73
2852.72	C–H stretching from alkanes	C–H stretching from alkanes	2873.94
-		O=C=O stretching from carbon dioxide	2358.94
-		O=C=O stretching from carbon dioxide	2341.58
1712.79	C=O stretching from α,β-unsaturated esters; aliphatic ketones; or carboxylic acids	C=O stretching from esters (6-membered lactone); δ lactone or aldehydes	1732.08
1654.92	C=O stretch from primary amides or δ lactame/ C=C stretching from alkenes	C=O stretch from primary amides or δ lactame/ C=C stretching from alkenes	1654.92
1602.85	C=C stretching from conjugated or cyclic alkenes	C=C stretching from conjugated or cyclic alkenes	1600.92
1516.05	C=C stretch from aromatic compounds	C=C stretch from aromatic compounds	1516.05
1463.97	C–H bending from alkanes (methylene group)	C–H bending from alkanes (methylene group)	1469.76
1377.17	O–H bending from phenols, alcohols, or carboxylic acids	O–H bending from phenols, alcohols, or carboxylic acids	1377.17
1261.45	C–O stretching from aromatic esters, ethers, acids, or alkyl aryl ether	C–O stretching from aromatic esters, ethers, or acids	1280.73
1165.00	C–O stretching from esters or tertiary alcohols	C–O stretching from esters or tertiary alcohols	1165.00
-		C–O stretching from aliphatic ethers or secondary alcohols	1118.71
1087.85	C–O stretching from primary alcohols	C–O stretching from primary alcohols	1072.42
1035.77	C–O stretching from esters		-
-		C=C bending from alkanes (monosubstituted) or =C–H bending from alkenes	981.77
-		=C–H bending from alkenes	947.05
-		=C–H bending from alkenes	912.33
815.89	C=C bending from alkanes	C=C bending from alkanes	856.39
-		C=C bending from alkanes	798.53
721.38	C=C bending from alkenes (disubstituted (cis)) or C–Cl stretching from halo compounds	=C–H bending from alkenes C–Cl stretching from halo compounds	742.59
-		=C–H bending from alkenes C=C bending from alkenes (disubstituted (cis))	696.30
-		C–Br stretching from halo compounds	650.01
597.93	C–I stretching from halo compounds	C–I stretching from halo compounds	578.64
516.92	C–I stretching from halo compounds		-

**Table 2:** The content of the polyphenolic compounds identified through LC-MS analysis.

GvDEE			
Compound Name	UV Identified	MS Qualitatively Identified	Concentration [μg/mL]
Chlorogenic acid	No	Yes	-
p-Coumaric acid	Yes	Yes	0.381
Ferulic acid	Yes	Yes	0.658
Isoquercitrin	Yes	Yes	2.970
Rutin	Yes	Yes	0.560
Quercetol	Yes	Yes	14.653
Luteolin	Yes	Yes	2.403
Apigenin	Yes	Yes	0.579
GvBuOH			
Chlorogenic acid	No	Yes	-
Isoquercitrin	Yes	Yes	2.508
Rutin	Yes	Yes	2.343

**Table 3:** Content of catechins found in *G. verum* extracts.

Extract	Concentration [μg/mL]					
	Epicatechin	Catechin	Syringic Acid	Gallic Acid	Protocatechuic Acid	Vanillic Acid
GvDEE	0.88	ND	ND	ND	ND	ND
GvBuOH	ND	ND	ND	ND	ND	ND

ND—not detected.

**Table 4:** The antioxidant capacity [%] of the *G. verum* extracts at different concentrations tested as compared with standard (Vit C) and the corresponding EC_50_ values, respectively.

Samples Concentration Tested [mg/mL]	Antioxidant Capacity [%]			EC_50_ [mg/mL]	
	Standard (Vit C)—0.4 mg/mL	GvDEE	GvBuOH	GvDEE	GvBuOH
1	97.08 ± 0.04	91.11 ± 0.04	81.64 ± 0.06	0.12 ± 0.03	0.18 ± 0.05
0.8		84.64 ± 0.04	70.03 ± 0.04		
0.5		69.11 ± 0.04	57.49 ± 0.06		
0.3		55.97 ± 0.04	48.94 ± 0.04		
0.1		43.55 ± 0.03	41.76 ± 0.03		
0.05		40.47 ± 0.01	38.92 ± 0.04		

The results are expressed as average ± SD (n = 3).

**Table 5:** The minimum inhibitory concentration (MIC) and the minimum bactericidal concentration (MBC) values.

*G. verum* Extracts	Microbial Strains	MIC [mg/mL]	MBC [mg/mL]
GvDEE	*Streptococcus pyogenes* (Gram+)	15	15
	*Staphylococcus aureus* (Gram+)	15	15
	*Escherichia coli* (Gram−)	30	30
	*Pseudomonas aeruginosa* (Gram−)	NA	NA
GvBuOH	*Streptococcus pyogenes* (Gram+)	NA	NA
	*Staphylococcus aureus* (Gram+)	NA	NA
	*Escherichia coli* (Gram−)	NA	NA
	*Pseudomonas aeruginosa* (Gram−)	NA	NA

NA—no antimicrobial activity.

## Data Availability

The data presented in this study are available on request from the corresponding author.
